# Identification of Two Tyrosine Residues Required for the Intramolecular Mechanism Implicated in GIT1 Activation

**DOI:** 10.1371/journal.pone.0093199

**Published:** 2014-04-03

**Authors:** Antonio Totaro, Veronica Astro, Diletta Tonoli, Ivan de Curtis

**Affiliations:** 1 Cell Adhesion Unit, Division of Neuroscience, San Raffaele Scientific Institute, Milano, Italy; 2 Università Vita-Salute San Raffaele, Milano, Italy; Imperial College London, United Kingdom

## Abstract

GIT1 is an ArfGAP and scaffolding protein regulating cell adhesion and migration. The multidomain structure of GIT1 allows the interaction with several partners. Binding of GIT1 to some of its partners requires activation of the GIT1 polypeptide. Our previous studies indicated that binding of paxillin to GIT1 is enhanced by release of an intramolecular interaction between the amino-terminal and carboxy-terminal portions that keeps the protein in a binding-incompetent state. Here we have addressed the mechanism mediating this intramolecular inhibitory mechanism by testing the effects of the mutation of several formerly identified GIT1 phosphorylation sites on the binding to paxillin. We have identified two tyrosines at positions 246 and 293 of the human GIT1 polypeptide that are needed to keep the protein in the inactive conformation. Interestingly, mutation of these residues to phenylalanine did not affect binding to paxillin, while mutation to either alanine or glutamic acid enhanced binding to paxillin, without affecting the constitutive binding to the Rac/Cdc42 exchange factor βPIX. The involvement of the two tyrosine residues in the intramolecular interaction was supported by reconstitution experiments showing that these residues are important for the binding between the amino-terminal fragment and carboxy-terminal portions of GIT1. Either GIT1 or GIT1-N tyrosine phosphorylation by Src and pervanadate treatment to inhibit protein tyrosine phosphatases did not affect the intramolecular binding between the amino- and carboxy-terminal fragments, nor the binding of GIT1 to paxillin. Mutations increasing the binding of GIT1 to paxillin positively affected cell motility, measured both by transwell migration and wound healing assays. Altogether these results show that tyrosines 246 and 293 of GIT1 are required for the intramolecular inhibitory mechanism that prevents the binding of GIT1 to paxillin. The data also suggest that tyrosine phosphorylation may not be sufficient to release the intramolecular interaction that keeps GIT1 in the inactive conformation.

## Introduction

During cell migration the intracellular pathways activated in response to extracellular motogenic stimuli need to be spatially and temporally regulated to ensure the synchronization of complex activities such as adhesion, cytoskeleton remodeling, and membrane traffic. Several studies have indicated a role of GIT proteins in cytoskeletal rearrangement and focal adhesion dynamics during motility in distinct cellular contexts, from migrating cells to neurons [1]–[3]. The members of the GIT family, GIT1 and GIT2, combine the ArfGAP enzymatic activity toward Arf GTPases with the scaffolding function in a variety of signalling complexes [1], [3]. GIT proteins include an N-terminal ArfGAP domain to inactivate Arf GTPases *in vitro* [4], and Arf6 in cells [1], a well known regulator of membrane traffic during cell migration [5]. Adjacent to the ArfGAP domain are three ankyrin repeats and a Spa2-homology domain (SHD), a tandem repeat of a sequence homologous to the yeast Spa2. Spa2 is a scaffold-like protein of a polarizing complex involved in actin cytoskeleton reorganization [6]. The SHD domain is important for binding to the PIX proteins [7]–[10]. The C-terminus of GIT1 contains a leucine zipper domain involved in homodimerization [11]–[13], and a paxillin binding sequence (PBS) [14]. GIT1 and paxillin are positive regulators of protrusion at the leading edge of migrating cells, and the recruitment of paxillin to focal adhesions is necessary for their turnover [15]. Paxillin participates in the recruitment of GIT/PIX complexes to focal adhesions by the interaction of the LD4 domain of paxillin with the PBS of GIT [16]–[20].

The members of the PIX family of Rac/Cdc42 guanine exchange factors are stable partners of GIT1 [7], [8], [12], [13], [21], while binding of GIT1 to either paxillin or the adaptor protein liprin-α1 is regulated [10], [22]–[23]. The mechanism regulating the binding to either paxillin or liprin-α1 is poorly understood. Our previous work indicated that GIT1 is regulated by an intramolecular inhibitory mechanism, and that PAK may act as an activator of GIT1 by inducing the release of the autoinhibitory interaction [23]. The release of this intramolecular interaction leading to increased binding of GIT1 to paxillin was obtained either by N-terminal truncation or by deletion of specific internal domains of GIT1. The previous biochemical and functional analysis indicated that the intramolecular inhibitory mechanism relies on the interaction between the N- and C-terminal portions of GIT1. Deletion of either the N-terminal ArfGAP or ankyrin domains, as well as the deletion of the C-terminal SHD domain is sufficient to abolish the intramolecular interaction, to increase binding to paxillin, and to affect cell spreading and morphology [23]. Here, we have addressed the mechanisms mediating the intramolecular interaction, and identified two tyrosine residues important to keep the GIT1 protein in the inactive state. Mutation of these residues activates GIT1 binding to paxillin and positively affects cell motility.

## Materials and Methods

### Plasmids

Plasmids encoding GFP-tagged human GIT1 (770 amino acid residues, NP 001078923.1) [19] and the mutants GFP-GIT1-Y392E, GFP-GIT1-Y554E, GFP-GIT1-S422D/S423D, GFP-GIT1-S457D/S458D, GFP-GIT1-S507D/T508D, GFP-GIT1-S545D/T546D were kindly provided by Alan Rick Horwitz (University of Virginia, Charlottesville, VA). Plasmids GPF-GIT1-Y246E, GFP-GIT1-Y293E, GFP-GIT1-Y246E/Y293E, GFP-GIT1-Y246A/Y293A, and GFP-GIT1-Y246F/Y293F were obtained by site directed mutagenesis starting from GFP-GIT1. Plasmids GFP-GIT1-N, GFP-GIT1-N-Y246F/Y293F and GFP-GIT1-N-Y246E/Y293E were obtained by PCR amplification of the corresponding GFP-GIT1 full length constructs with the primers 5′-cgGAATTCcATGTCCCGAAAGGGGCCGC-3′ and 5′-cgcGGATCCTTAGTCGATGATCAAGGTGGCAAAC-3′. The PCR products were digested with EcoR I and BamH I restriction enzymes and cloned in the pEGFP-C1 vector digested with the same enzymes. The constructs FLAG-GIT1 wildtype, FLAG-GIT1-FF and FLAG-GIT1-EE were obtained by digesting the corresponding pEGFP human GIT1 constructs with EcoRI and XmaI enzymes, and subcloning the resulting fragments in pFLAG CMV2 vector digested with the same enzymes. Plasmids FLAG-GIT1-N (residues 1–346), FLAG-GIT1-N2 (residues 1–230), FLAG-GIT1-C (residues 346–740), FLAG-GIT1-C2 (residues 229–740) and FLAG-GIT1-C2-LZ were as described [8], [12], [23]. The plasmid HA-GIT1-Spa-I (residues 1–321) was obtained by removing with the restriction enzyme ScaI the fragment coding for the carboxy-terminal portion of GIT1 from a pBK-HA chicken GIT1 plasmid. The plasmid encoding the GFP-GIT1-Y246E/Y293E-ΔPBS mutant lacking the region of interaction with paxillin (residues 624–770) was obtained by digesting the GFP-GIT1-Y246E/Y293E plasmid with XbaI and by intra-molecular religation. The orange-paxillin plasmid was provided by Alan Rick Horwitz (University of Virginia, Charlottesville, VA).

### Antibodies

Monoclonal antibodies (mAbs) against FLAG (clone M2), and polyclonal antibodies (pAbs) against FLAG or mouse IgG were from Sigma-Aldrich (St. Louis, MO). The anti-haemagglutinin mAb (HA, clone 12CA5) was from PRIMM (Milan, Italy). The anti-paxillin mAb (clone 349) was from BD Biosciences (San Jose, CA). The anti-GIT1 pAb raised in goat, was from Santa Cruz Biotechnology (Santa Cruz, CA). The anti-phosphotyrosine mAb (Clone 4G10) was from Millipore (Billerica, MA). The anti-Src mAb (Clone 327) was generously provided by Sara Courtneidge (Sanford Burnham Medical Research Institute, La Jolla, CA). The pAb against GFP, and Alexa-488 anti-rabbit secondary antibodies were from Molecular Probes, Invitrogen. Tha anti-βPIX antibody was described previously (**12**).

### Cell Culture and Transfection

COS7 cells were cultured in DMEM containing 10% FetalClone III (Hyclone PERBIO, Erembodegem, Belgium). Cells seeded in 60 mm diameter dishes were transfected with Lipofectamine-2000 (Invitrogen). Cells were processed after 18–24 h.

### Biochemical Analysis

Cells washed twice with ice-cold TBS (20 mM Tris-HCl, pH 7.5, 150 mM NaCl) were lysed in lysis buffer (0.5% Triton X-100, 150 mM NaCl, 20 mM Tris-Cl, 2 mM MgCl2, 1 mM sodium orthovanadate, 10 mM sodium fluoride; pH 7.5) in the presence of anti-proteases (1× Complete, Roche, Manheim, Germany). The insoluble material was removed by centrifugation for 15 min at 12000 g at 4°C. Protein determination was done using the Bradford protein assay reagent from BIO-RAD (Hercules, CA, USA).

When indicated, cells were incubated for 20 min at 37°C with 1 mM pervanadate before lysis, to inhibit protein tyrosine phosphatases and increase the levels of protein phosphorylation. Pervanadate was prepared immediately before use by mixing 1 M Na_3_VO_4_ and 30% H_2_O_2_ to obtain a 100 mM Na_3_VO_4_, 100 mM H_2_O_2_ solution. After 30 min at 37°C, 0.2 mg/ml catalase was added and incubated for 30 min at 37° to remove H_2_O_2_.

For immunoprecipitation, primary Abs were pre-adsorbed to protein A Sepharose beads (Amersham Biosciences). The anti-paxillin mAb was adsorbed to the protein A Sepharose beads preadsorbed with rabbit anti-mouse IgG. Beads were incubated with aliquots of lysate for 2 h at 4°C. After washing with lysis buffer with 0.1% Triton X-100, immunoprecipitates, lysates and unbound fractions were analyzed by immunoblotting.

### Haptotactic Migration Assay

COS7 cells transfected with the indicated GFP-GIT1 constructs were starved overnight in serum-free medium (DMEM with 0.1% bovine serum albumin). Transfected cells were trypsinized, resuspended in serum-free medium and seeded in transwell (40000 cells/transwell; 8 μm pore PET membrane from Millipore, Billerica, MA). The lower side of the membrane was coated with 10 μg/ml of fibronectin. After 6 h at 37°C non-migrating cells were removed from the upper chamber, and cells on the lower side were fixed with 3% paraformaldehyde and detected by fluorescence. For quantification, GFP-positive cells were counted from different representative fields per well. Control fields with less than 30 cells were not considered. Data were collected from 2–3 independent experiments, each in duplicate. Values of migrated cells were normalized with respect to the percentage of transfected cells (40–60% transfection efficiency). Data are presented as normalized mean values ±SEM. Significance (<0.05%) was established by the Student’s t test.

### Wound Healing Assay

COS7 cells were seeded on 6-well plates coated with fibronectin (2.5 μg/ml) and cultured for one day before transfection. Transfected cells were starved overnight (0.5% BSA in DMEM) and scratched using a sterile p10 pipet tip. During time-lapse imaging cells were cultured in DMEM with 0.5% FCIII. Images were acquired for 18 h (1 frame every 7 min) with a Zeiss Axiovert S100 TV2 microscope (Oberkochen, Germany) equipped with a Hamamatsu Orca II CCD digital camera from Hamamatsu Photonics K.K. (Hamamatsu, Japan), and a Cage Incubator designed to maintain the required environmental conditions. Migration paths were calculated with ImageJ based on the nuclear position of cells in 4 fields per well.

## Results and Discussion

### Specific Mutations in the Amino-terminal Half of GIT1 Enhance Binding to Paxillin

**Figure 1:**
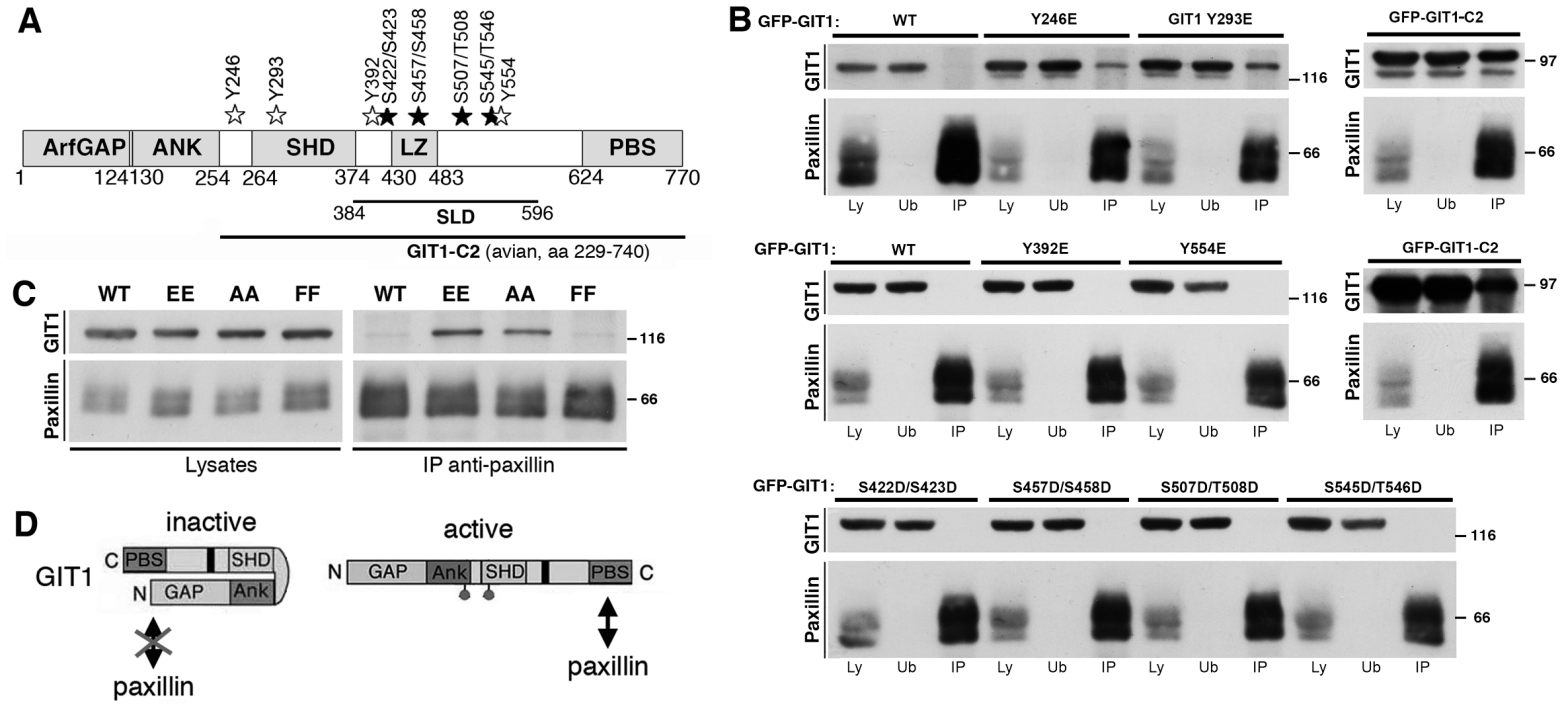
Mutations Y246E and Y293E of GIT1 enhance binding to paxillin. (**A**) Schematic representation of human GIT1 (NP 001078923.1). GFP-tagged wild type GIT1 was used to introduce phosphomimetic mutations. Tyrosine (Y) or serine (S) and threonine (T) residues were mutated into glutamic acid or aspartic acid, respectively. White and black stars indicate the locations of the mutated tyrosine and serine/threonine residues, respectively. ArfGAP, Arf GTPase-activating protein; ANK, ankyrin repeats; SHD, Spa2-homology domain; LZ, leucine zipper; PBS, paxillin binding site; SLD, synaptic localization domain. (**B**) Aliquots of lysates (400 μg) from cells transfected with the indicated constructs were used for immunoprecipitation of endogenous paxillin. Filters with immunoprecipitates (IP), and equal amounts (80 μg) of the respective lysates (Ly) or unbound fractions after immunoprecipitation (Ub) were blotted with anti-GFP (for GIT1) or anti-paxillin antibodies. Molecular weight markers are indicated to the right of each blot. (**C**) The double substitution of residues Y246 and Y293 with either two glutamic acid (EE) or two alanine residues (AA) enhanced GIT1 binding to paxillin. Aliquots of lysates (200 μg protein) from cells transfected with the indicated constructs were immunoprecipitated with anti-paxillin antibody. Filters with immunoprecipitates (IP), and lysates (40 μg) were blotted as indicated, using anti-GFP (for GIT1) or anti-paxillin antibodies. (**D**) Model for GIT1 activation: see details in the text.

We have previously shown that either endogenous or overexpressed GIT1 binds very poorly to endogenous paxillin, while deletion of different segments of the amino-terminal portions of the polypeptide induces strong binding of paxillin to the carboxy-terminal portion of the truncated proteins [23]. To limit the possible effects of these deletions on the overall structure of the GIT1 protein, we have tested here the hypothesis that activation may be obtained by site-directed mutagenesis of specific residues of GIT1. A number of studies indicate that GIT1 is phosphorylated in response to different stimuli [7], [24]–[26], and that phosphorylation may modulate GIT1 functions in different cells [14], [27]–[28]. For this reason, we have considered several residues that have been shown to be sites of phosphorylation on the GIT1 proteins (**Figure 1A** **, Figure S1**) [29], and tested whether one or more of these sites were able to regulate GIT1 function measured as its ability to bind paxillin.

Specific point mutations were introduced into the GFP-GIT1 wildtype construct. In particular, tyrosine, or serine/threonine residues were mutated, in glutamic acid or aspartic acid respectively, to mimic the phosphorylated states of these residues. To limit this analysis, we excluded tyrosine residues located in the PBS region, since their phosphorylation does not appear to be involved in the regulation of GIT1-paxillin interaction [14]. Conversely, residues Y246 and Y293 were selected for being the only two phosphorylated tyrosines in the amino-terminal portion of GIT1 [29]. The phosphorylation sites of GIT1 located in the central part of the protein (SLD, synaptic localization domain [29]–[30]) were also considered for the analysis, including the two tyrosines Y392 and Y554, and the serine/serine or serine/threonine couples S422/S423, S457/S458, S507/T508, and S54/T546. The mutations introduced in the GFP-tagged full length protein were tested by immunoprecipitation to look at the effects on the binding of GIT1 to endogenous paxillin. We confirmed the very poor binding of endogenous paxillin to the full length GIT1 protein: immunoblotting on immunoprecipitates with anti-paxillin antibodies performed on lysates of COS7 cells transfected with GFP-tagged GIT1 constructs showed virtually no binding of wildtype GIT1 to endogenous paxillin, while paxillin interacted with the truncated GIT1-C2 protein (**Figure 1B**). Similarly, several mutants of either tyrosine or serine/threonine residues with glutamic acid or aspartic acid residues respectively (tyrosine mutants GIT1-Y392E and GIT1-Y554E; serine/threonine mutants GIT1-S422D/S423D, GIT1-S457D/S458D, GIT1-S507D/T508D, and S545D/T546D) showed virtually no binding to paxillin. In contrast, mutation of either Y246 or Y293 with glutamic acid (GIT1-Y246E and GIT1-Y293E respectively) resulted in a clear interaction with endogenous paxillin (**Figure 1B**).

The exchange of either serine or threonine residues with aspartic acid or glutamate residues are considered phosphomimetic substitutions. The exchange of a tyrosine with a glutamate has also been considered phosphomimetic in several studies. On the other hand, apart from the negative charge, phosphotyrosine is very different from glutamate, since it is shorter than phosphotyrosine and lacks an aromatic ring. Therefore we have further challenged the hypothesis that the effects of the substitution of Y246 and Y293 by glutamate residues on the binding of GIT1 to paxillin could be due to the phosphomimetic effects of these mutations. The double mutation of Y246 and Y293 with two glutamates enhanced the binding of GIT1-Y246E/Y293E to endogenous paxillin (**Figure 1C**). Surprisingly, also the substitution of the two tyrosines with alanine residues induced a clear binding of the GIT1-Y246A/Y293A mutant to paxillin, indicating that the change of the biochemical properties observed for these GIT1 mutants was not due to the phosphomimetic effect of the glutamate substitution. Intriguingly, the GIT1-Y246F/Y293F mutant where the two tyrosines were substituted by two phenylalanines behaved as the wildtype protein, since only background binding to paxillin could be detected to this mutant. Altogether these results show that specific point mutations in the region of GIT1 between the ankyrin repeats and the SHD are sufficient to affect the ability of GIT1 to bind paxillin.

Our previous data support the existence of two conformation of the GIT1 polypeptide [23]: a close conformation where the amino-terminal portion of GIT1 interacts with its carboxy-terminal part, and an open conformation that allows the binding of paxillin to the carboxy-terminal part of the polypeptide. In this model the change in the efficiency of association of GIT1 with paxillin is considered as a measure of the release of the intramolecular interaction. Here, the results obtained with the GIT1 mutants fit with the proposed model of GIT1 activation (**Figure 1D**). The data presented suggest that specific mutations act by allowing the carboxy-terminal PBS to become available for binding to paxillin. Interestingly, the two residues Y246 and Y293, which if mutated alter the biochemical properties of the GIT1 protein, are located in the ankyrin and SHD regions that are necessary to keep the protein in a closed binding-incompetent conformation [23]. On the other hand, substitution of the two tyrosines with phenylalanines that preserve the aromatic ring found also in the tyrosine, maintain the GIT1 protein in the inactive conformation, indicating that the hydrophobic and/or bulky properties of these residues are important for keeping GIT1 in the inactive state.

### The Mutations of Tyrosines 246 and 293 do not Affect Binding of GIT1 to βPIX

**Figure 2:**
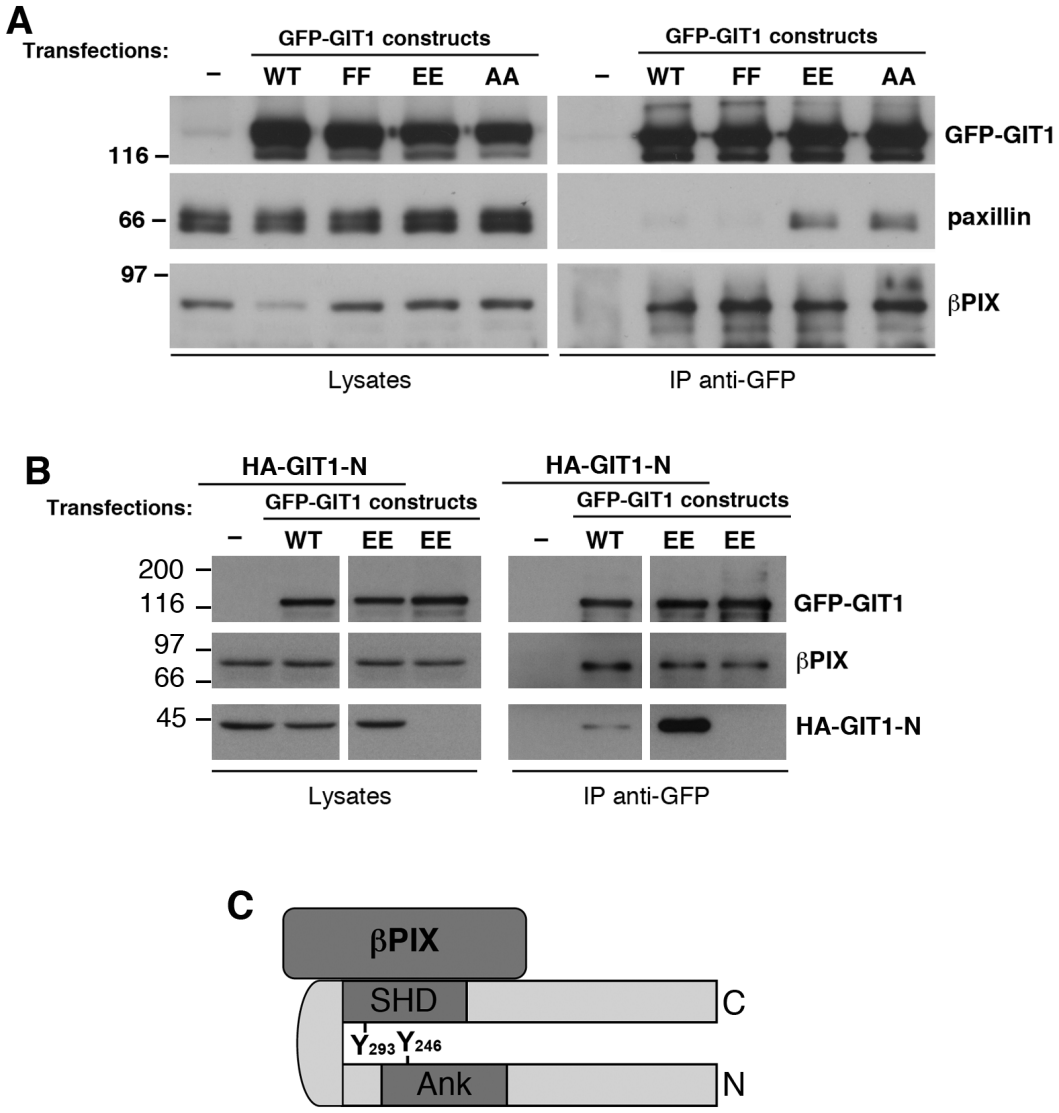
Mutation of tyrosines 246 and 293 does not affect the binding of GIT1 to βPIX. (**A**) Lysates from mock-transfected cells or from cells transfected with the indicated GIT1 constructs were immunoprecipitated for GIT1 with anti-GFP antibody. Filters with immunoprecipitates (IP, from 200 μg of protein lysate), and lysates (25 μg) were blotted with anti-GFP (for GIT1), anti-paxillin, and anti-βPIX antibodies. (**B**) Binding of GIT1-N to GIT1-Y246E/Y293E does not affect the interaction of GIT1-Y246E/Y293E to βPIX. Lysates from COS7 cells co-transfected to express the HA-GIT1-N fragment and the full length GFP-GIT1-WT or GIT1-Y246E/Y293E proteins were immunoprecipitated (IP, from 175 μg of protein lysate) with anti-GFP. Lysates (25 μg, oƒn the lef) and IP (right) were blotted to reveal the full length proteins (anti-GFP), the HA-GIT1-N fragment (anti-HA mAb 12CA5), and endogenous βPIX. (**C**) Our data support the hypothesis that the tyrosine 293 of the SHD domain of GIT1 is required for the intramolecular interaction, but not for the interaction of the SHD domain of GIT1 with βPIX.

We have tested if the interaction of GIT1 with other binding partners was affected by mutation of tyrosines 246 and 293. Our previous results have indicated that GIT1 forms stable complexes with the Rac/Cdc42-specific exchanging factor βPIX [23]. The βPIX protein interacts with GIT1 by binding to the SHD domain. We have tested whether the mutations of tyrosines 246 and 293 had an effect on the binding of βPIX to GIT1. We found that binding to endogenous βPIX was similar for wildtype, GIT1-Y246E/Y293E, GIT1-Y246A/Y293A, and GIT1-Y246F/Y293F mutant proteins (**Figure 2A**). These results show that the modification in the structure of GIT1 introduced by the mutation of the two tyrosines specifically affects the exposure of the paxillin binding region, without affecting the stable association between GIT1 and PIX. Moreover, binding of endogenous βPIX to the active GIT1-EE mutant was not affected by the interaction of GIT1-Y246E/Y293E with the GIT1-N fragment (**Figure 2B**). These data indicate that GIT1-N does not compete with βPIX for binding to GIT1-Y246E/Y293E. Since tyrosine 293 is in the SHD domain required for binding to βPIX, the results also support the hypothesis that this tyrosine residue is required for the intramolecular interaction, but not for the interaction of GIT1 with βPIX (**Figure 2C**).

### Tyrosines 246 and 293 are Required to Keep GIT1 in a Closed Conformation

**Figure 3:**
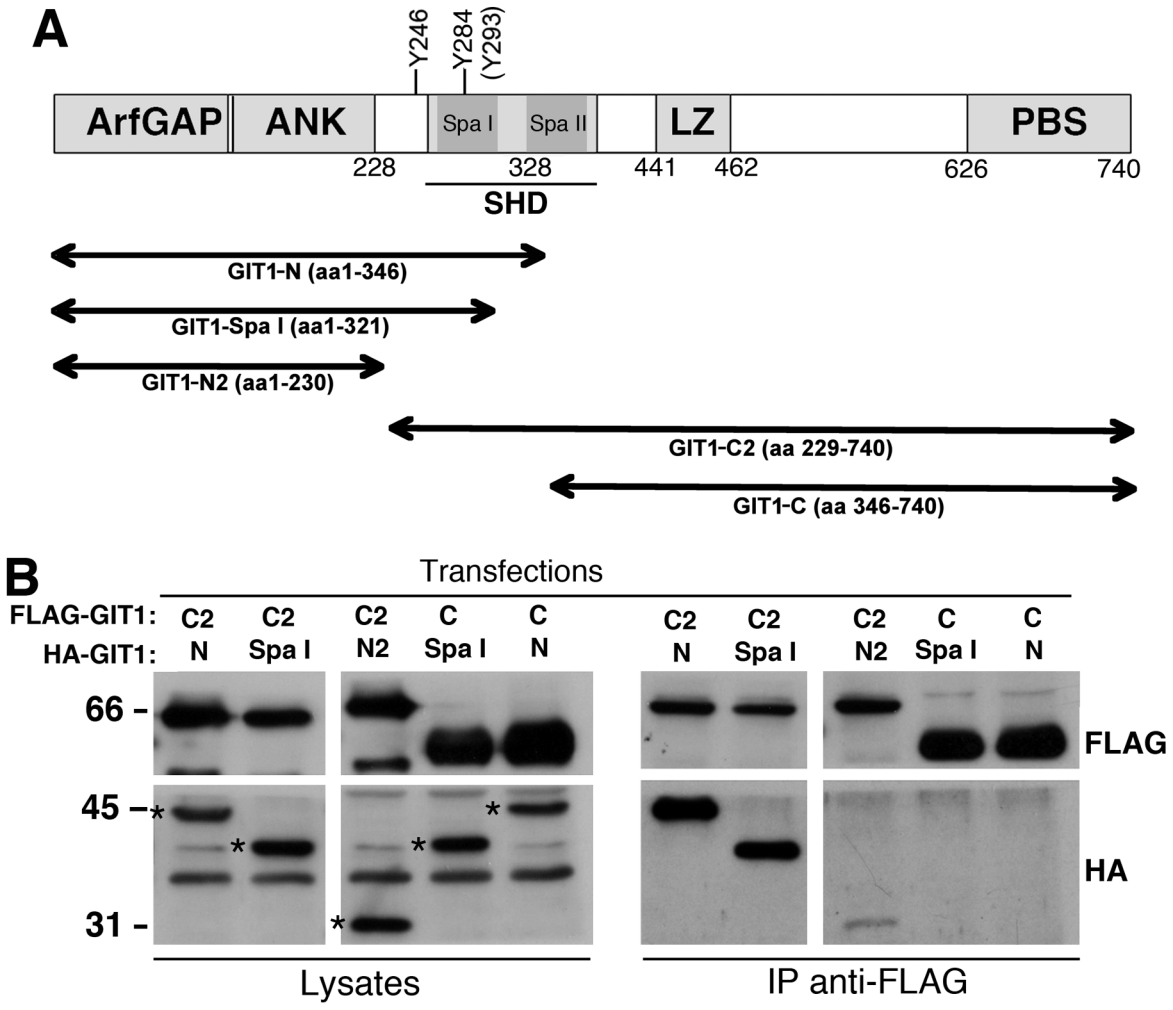
Efficient binding of the amino-terminal portion of GIT1 to the carboxy-terminal portion requires the inclusion of the first Spa2 region of the SHD. (**A**) Scheme of the constructs of avian GIT1 used in the following experiment. To be noted that tyrosine 284 of avian GIT1 corresponds to tyrosine 293 of human GIT1. (**B**) COS7 cells were cotransfected with the indicated combination of HA-tagged amino-terminal and FLAG-tagged carboxy-terminal fragments of GIT1. Lysates were immunoprecipitated with the anti-FLAG M2-conjugated beads. Filters with immunoprecipitates (IP, from 250 μg of protein lysate) and lysates (50 μg) were incubated with anti-HA or anti-FLAG antibodies. Amino-terminal fragments in the lysates are indicated by asterisks.

The model proposed for the activation of the GIT1 protein (**Figure 1D**) is based on a set of experiments with fragments of the GIT1 protein [23]. We confirmed our previous findings that the GIT1-N fragment interacts efficiently with the carboxy-terminal GIT1-C2 fragment, but not with GIT1-C, a carboxy-terminal fragments lacking an intact SHD domain (**Figure 3** **, Figure S1**). The GIT1-N2 fragment including the three ankyrin repeats, but lacking the SHD domain interacted only weakly with GIT1-C2. On the other hand, the GIT1-SpaI fragment including the first Spa2 repeat of the two forming the SHD domain can interact efficiently with GIT1-C2, but not with GIT1-C. These data suggest that inclusion of the two tyrosine residues 246 and 293 in the amino-terminal portion of GIT1 is important for the efficient interaction with the carboxy-terminal portion of the GIT1 polypeptide.

**Figure 4:**
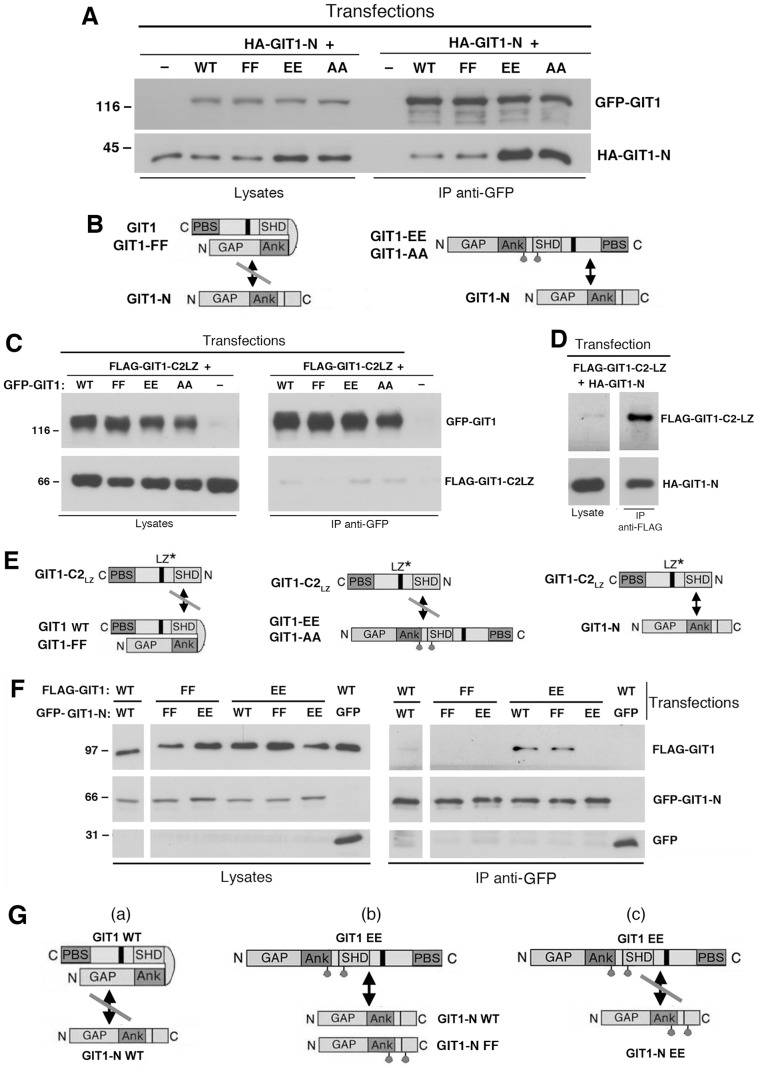
Tyrosines 246 and 293 are required to hold GIT1 in a closed conformation. (**A,C,D,F**) COS7 cells were cotransfected with the indicated constructs, lysed, and immunoprecipitated with anti-GFP (**A,C,F**) or anti-FLAG antibodies (**D**). Filters with immunoprecipitates (IP) and lysates were blotted to reveal the full length GFP-tagged constructs (with anti-GFP), HA-GIT1-N (with mouse 12CA5 mAb), or GIT1-C2-LZ fragment (with anti-FLAG). (**B,E,G**) Schemes of the interactions proposed from the experiments presented in (**A**), (**C,D**), and (**F**), respectively. The asterisks in (**E**) indicate the monomeric mutated carboxy-terminal fragment that can not dimerize. See the Results for details.

To further explore this hypothesis, we have verified if the introduction of specific point mutations in the full length GIT1 polypeptide was sufficient to induce the opening of the full length protein, thus making it available for the interaction with an exogenous amino-terminal portion (GIT1-N). As expected, the amino-terminal GIT1-N fragment interacted poorly with the wildtype GIT1 protein (**Figure 4A**). On the other hand, GIT1-N interacted more strongly with the full length double mutants GIT1-Y246E/Y293E and GIT1-Y246A/Y293A, but not with GIT1-Y246F/Y293F, indicating that the mutation of the two tyrosines to either alanine or glutamate was sufficient to unlock the closed conformation of the GIT1 polypeptide (**Figure 4B**), thus making its carboxy-terminal portion available to the binding with the supplied amino-terminal GIT1-N fragment.

Next we tested the hypothesis that the two amino-terminal tyrosines 246 and 293 are required for the binding of the amino-terminal portion of the full length GIT1 to an exogenous carboxy-terminal GIT1 fragment. For this, we compared the interaction of the monomeric carboxy-terminal construct GIT1-C2-LZ (obtained by mutating two leucine residues in the carboxy-terminal leucine zipper to prevent homodimerization, [12]) with either wildtype GIT1 or with the full length tyrosine mutants. Interestingly, neither GIT1, nor any of the three mutants GIT1-Y246E/Y293E, GIT1-Y246A/Y293A, and GIT1-Y246F/Y293F were able to bind the carboxy-terminal GIT1-C2-LZ fragment (**Figure 4C**). As a positive control, GIT1-C2-LZ could bind efficiently the amino-terminal fragment GIT1-N including the tyrosines 246 and 293 (**Figure 4D**). These results indicate that the two tyrosines in the amino-terminal part of the GIT1 polypeptide are needed for the interaction with the carboxy-terminal portion of the protein to keep the polypeptide in the closed conformation (**Figure 4E**): the mutant proteins with an open conformation (GIT1-Y246E/Y293E, GIT1-Y246A/Y293A) can not interact with the carboxy-terminal fragment due to the lack of the proper amino-terminal residues; on the other hand in the proteins with a closed conformation (GIT1-Y246F/Y293F and wildtype GIT1) the proper residues are not available because already occupied in intramolecular interactions (**Figure 4E**).

To further test this model, we analyzed the binding of wildtype and mutant GIT1-N fragments to either wildtype or mutant full length GIT1 polypeptides. We prepared GIT1-N fragments including the substitutions of the two tyrosines 246 and 293 with either glutamate or phenylalanine residues. Cells cotransfected with different combinations of FLAG-tagged full length and GFP-tagged amino-terminal fragment of GIT1 were immunoprecipitated with anti-GFP antibodies and analyzed by immunoblotting to detect the coprecipitation of the full length FLAG-GIT1 proteins with GFP-GIT1-N. As expected, the full length wildtype GIT1 protein did not interact with the wildtype GIT1-N fragment, while the full length “activated” GIT1-Y246E/Y293E double mutant interacted with either wildtype GIT1-N, or the mutated GIT1-N-Y246F/Y293F, but not with the GIT1-N-Y246E/Y293E fragment (**Figure 4F**). On the other hand, the full length GIT1-Y246F/Y293F mutant did not interact with either GIT1-Y246E/Y293E or GIT1-N-Y246F/Y293F fragments.

These data demonstrate that opening of the full length protein by dysrupting the intramolecular interaction mediated by Y246 and Y293 allows binding of the exposed carboxy-terminal part of the full length mutants to the exogenous wild type GIT1-N fragment (**Figure 4B**). On the other hand, the substitution of Y246 and Y293 in the GIT1-N fragment with two glutamates prevented the interaction with the exposed carboxy-terminus of the mutant full length protein (**Figure 4G** **, scheme c**). Conversely the interaction could still occur if the two tyrosines in GIT1-N were substituted by two phenylalanines (**Figure 4G** **, scheme b**). These findings support the hypothesis that the aromatic rings of tyrosines 246 and 293 are important for the intramolecular interaction between the amino- and carboxy-terminal portions of the GIT1 polypeptide that keeps the protein in the inactive state.

### Tyrosine Phosphorylation does not Prevent the Intramolecular Interaction Mediated by Y246 and Y293

**Figure 5:**
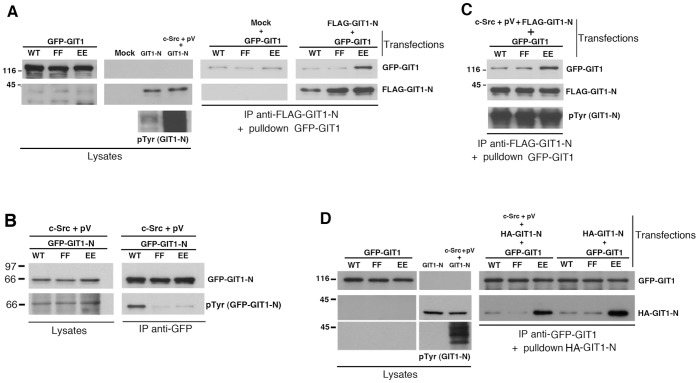
Hyperphosphorylation of GIT1-N by Src and pervanadate does not affect its binding *in vitro* to full length GIT1 proteins. (**A–C**) COS7 cells were transfected with full length GFP-GIT1 or GIT1-N constructs (WT, FF, or EE), or with wildtype or mutant FLAG-GIT1-N fragments, alone or together with c-Src. The cells cotransfected with c-Src were incubated 20 min at 37°C with 1 mM pervanadate before lysis (pV). Aliquots of the lysates (200 μg of protein) were immunoprecipitated (IP) with anti-FLAG (**A,C**) or anti-GFP (**B**) antibodies. For pulldowns shown in (**A**) and (**C**): FLAG-immunoprecipitates were washed and incubated for 2 h at 4°C with lysates (250 μg of protein) from cells transfected with the indicated full length GFP-GIT1 constructs. Equal amounts of lysates (25 μg of protein), and the pulldowns performed with GIT1-N without (**A**) or with c-Src and pervanadate treatment (**C**) were blotted for the detection of the indicated antigens. In (**B**) the immunoprecipitations with anti-GFP antibody (right) were immunoblotted to detect the levels of GFP-GIT1-N protein (upper filter) and of its tyrosine phosphorylation (lower filter). (**D**) COS7 cells were transfected to express the indicated GFP-GIT1 mutants, or transfected with the HA-GIT1-N fragment alone or together with c-Src. The cells co-transfected with c-Src were treated as in (**A,C**). Aliquots of the lysates from cells expressing GFP-GIT1 mutants (250 μg of protein) were immunoprecipitated with anti-GFP. Pulldowns: GFP-immunoprecipitates were washed and incubated for 2 h at 4°C with lysates (400 μg of protein) from cells transfected with HA-GIT1-N alone, or together with c-Src. Equal amounts of lysates (25 μg of protein), and the pulldowns were blotted for the detection of the indicated antigens.

We have shown that the substitution of the two amino-terminal tyrosines with two glutamic acid or alanine residues induced the interaction of the full length mutant proteins with the amino-terminal GIT1-N fragment (**Figure 4A,B**). This result was obtained by immunoprecipitating the full length GFP-tagged proteins with anti-GFP antibodies from cotransfected cells. We then used a pulldown assay to reconstitute *in vitro* the binding of the mutated full length protein to the GIT1-N fragment. For this, we transfected the cells with FLAG-GIT1-N, and immunoprecipitated the polypeptide with M2 anti-FLAG antibodies conjugated to beads. After removing the unbound material, the beads with the immunoprecipitates were incubated for 2 h at 4°C with lysates of cells transfected with either wildtype or mutated full length GFP-GIT1 proteins. Under these experimental conditions, we observed the reconstitution *in vitro* of the interaction between GIT1-N and the GIT1-Y246E/Y293E mutant, while no interaction of GIT1-N with either GIT1-Y246F/Y293F or wildtype GIT1 was detected (**Figure 5A**). This result shows that it is possible to reconstitute *in vitro* the interaction of an exogenous GIT1-N fragment with the carboxy-terminal part of the full length GIT1 made available by mutation of Y246 and Y293. Together with the previous findings, these results are consistent with an activation model in which Y246 and Y293 are required to keep GIT1 in an inactive state, by means of intramolecular interactions that hold together the amino- and carboxy-terminal portions of the protein.

It has been shown that tyrosines 246 and 293 of GIT1 are phosphorylated by overexpression of Src and pervanadate treatment [29]. Phosphorylation of GIT1-N was achieved by co-expression of c-Src obtained by transfecting the pSGT-c-Src plasmid [31] in combination with pervanadate treatment to inhibit protein tyrosine phosphatases, as previously described [29]. This procedure allowed us to confirm that either one or both tyrosines 246 and 293 are sites of phosphorylation. Indeed, phosphorylation of either GIT1-N-Y246E/Y293E or GIT1-N-Y246F/Y293F fragments was strongly reduced with respect to the wildtype GIT1-N including both tyrosines (**Figure 5B**). We tested the possibility that phosphorylation of the two tyrosines by c-Src- and pervanadate-enhanced phosphorylation of GIT1-N blocked its binding to the full length GIT1-Y246E/Y293E. For this, we performed *in vitro* pulldown experiments with immunoprecipitates of the phosphorylated FLAG-GIT1-N protein (**Figure 5C**). This treatment resulted in a strong increase of the tyrosine phosphorylation of the GIT1-N fragment (**Figure 5C**). After immunoprecipitation of the phosphorylated GIT1-N, we incubated the beads carrying the immunoprecipitates with lysates from cells transfected with either wild type or mutated full length GIT1 proteins. Phosphorylation did not prevent GIT1-N to bind the full length GIT1-Y246E/Y293E mutant more efficiently with respect to either wild type or GIT1-Y246F/Y293F full length proteins (**Figure 5C**). Similar results were obtained when the pulldown was reversed: lysates containing the phosphorylated GIT1-N fragment were incubated with immunoprecipitated full length wild type or mutant GIT1 proteins (**Figure 5D**). Both control and hyperphosphorylated GIT1-N bound more strongly to GIT1-Y246E/Y293E than to either the wild type or the GIT1-Y246F/Y293F full length proteins. These results suggest that phosphorylation of the aminoterminal tyrosines 246 and 293 may not be sufficient to prevent the binding to the carboxy-terminal region of GIT1.

### The Binding of Full Length GIT1 to Paxillin can be Reconstituted *in vitro*, and is not Affected by Phosphorylation

**Figure 6:**
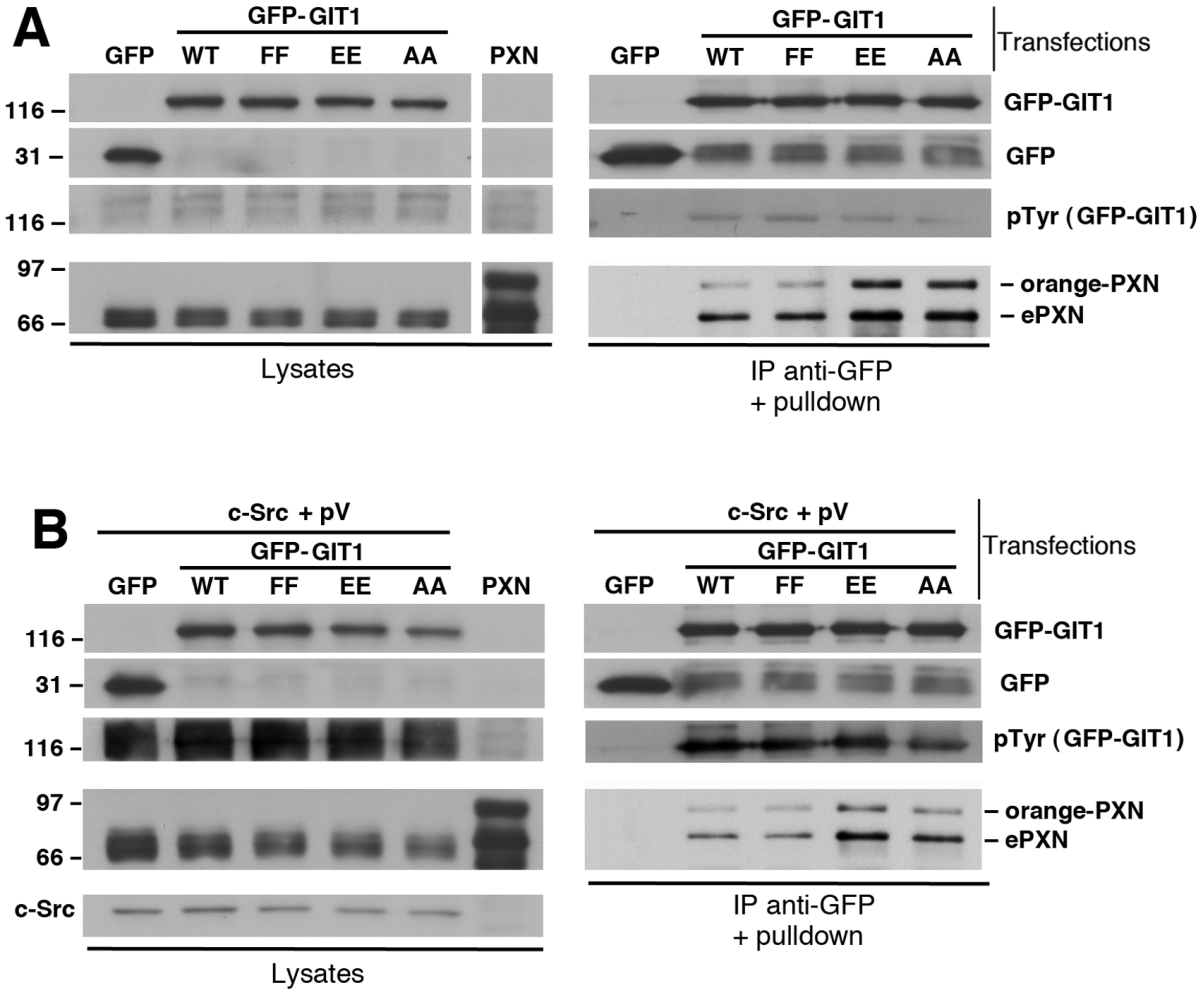
Effects of tyrosine phosphorylation on the binding of GIT1 to paxillin. COS7 cells were transfected with GIT1 constructs (**A**) or cotransfected with the GIT1 constructs together with c-Src (**B**). The cells cotransfected with c-Src were also treated for 20 min at 37°C with 1 mM pervanadate before lysis (pV). In parallel, a lysate was prepared from cells transfected with orange-paxillin (PXN). After immunoprecipitation of each GFP-GIT1 construct with anti-GFP antibodies (250 μg of protein lysate/immunoprecipitation), the beads were incubated for 2 h at 4°C with aliquots of the lysate containing orange-paxillin (180 μg). Lysates (25 μg) and immunoprecipitates (IP) after pulldown were used for immunoblotting for the indicated antigens.

We tested if the binding of active GIT1 forms to paxillin could be reconstituted *in vitro* (**Figure 6**). Cells were transfected with either GFP or with each of the wildtype or mutant full length GFP-GIT1 proteins. Lysates from the transfected cells were then used for pulldown experiments, by mixing the beads with the immunoprecipitated GFP-tagged proteins with aliquots of a lysate from cells transfected with orange-paxillin. The results show increased binding of endogenous paxillin and exogenous orange-paxillin to the full length GIT1-Y246E/Y293E and GIT1-Y246A/Y293A mutants compared to either wildtype GIT1 or GIT1-Y246F/Y293F (**Figure 6A**). These results indicate that the specific interaction between paxillin and the activated forms of the GIT1 can be reconstituted *in vitro.*

We next tested if tyrosine phosphorylation of the full length GIT1 polypeptides would affect the binding to paxillin *in vitro*. Cells were cotransfected with each of the wildtype or mutant full length GIT1 proteins together with the tyrosine kinase c-Src, and were incubated before lysis for 20 min with pervanadate, to inhibit protein phosphatases and enhance the levels of GIT1 phosphorylation. The combination of c-Src expression and pervanadate treatment strongly increased the tyrosine phosphorylation of the GIT1 proteins (**Figure 6B**). Pulldown assays were then performed as described in the previous paragraph. Binding of the activated forms of GIT1 to paxillin was not enhanced by c-Src- and pervanadate-enhanced tyrosine phosphorylation. In contrast, the data suggest that binding to the active forms of GIT1 (GIT1-Y246E/Y293E and GIT1-Y246A/Y293A) was moderately decreased compared to that observed after pulldown with the same constructs from cells where GIT1 was not hyperphosphorylated. This may be due to the phosphorylation of the carboxy-terminal portion of the GIT1 polypeptide that partially interferes with the binding to paxillin [14].

### GIT1-Y246E/Y293E Specifically Enhances Cell Motility

**Figure 7:**
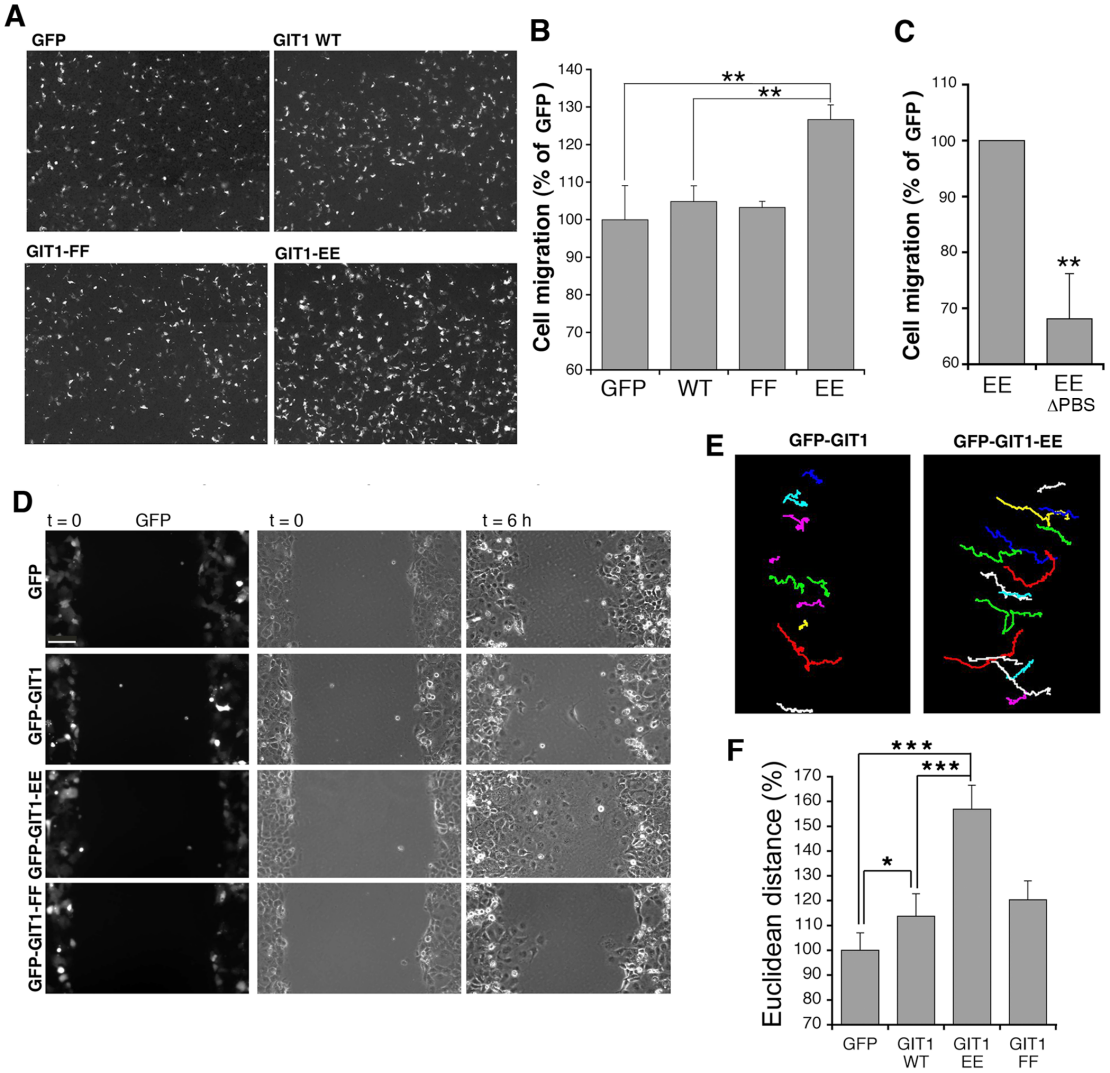
Expression of the “active” GIT1-Y246E/Y293E mutant increases the efficiency of migration. (**A**) COS7 cells transfected with GFP or with GFP-tagged GIT1 constructs were used for haptotactic cell migration towards fibronectin. Images show fields with cells migrated to the lower side of the filters coated with fibronectin. (**B,C**) Quantification of the number of cells migrated to the lower side of the wells. Bars are normalized mean values ±SEM from 12–19 fields from 2–3 independent experiments. **P<0.02. (**D–F**) Wound-healing assay. COS7 cells were transfected as in (**A**). Confluent monolayers were wounded and followed by time lapse imaging. (**D**) Fluorescent images (left column) showing GFP-positive transfected COS7 cells at time 0. The other columns show the phase contrast images of the same fields, at the beginning (t = 0) and the end (t = 6 h) of the assay. Bar, 100 μm. (**E**) Tracks of GFP-positive cells transfected with either GFP-GIT1 or GFP-GIT1-Y246E/Y293E. Tracks were taken during the wound healing assays and refer to an interval of 8 h. (**F**) Quantification of the euclidean distance covered by cells during the wound healing assay. Bars are mean values ±SEM from 90 cells from 3 different experiments. *P<0.05; ***P<0,002.

To test if the activation of the binding of GIT1 to paxillin reflects a functional outcome, we tested the effects of the overexpression of the different GIT1 full length constructs on cell migration. We used haptotactic cell migration with the lower side of the transwell membrane coated with fibronectin, to induce integrin-dependent migration of the cells plated on the upper chamber of the transwells. The results showed a significant increase (>25%) of the number of cells that migrated to the lower side of the filters only when the cells were transfected with the GIT1-Y246E/Y293E mutant (**Figure 7A,B**). This increase was significant with respect to either cells overexpressing GFP, or GFP-GIT1-WT. No effects were observed following overexpression of the GIT1-Y246F/Y293F mutant.

Deletion of the carboxy-terminal PBS of GIT1 prevents binding to paxillin [32]. Transfection with GIT1-Y246E/Y293E-ΔPBS, a GIT1-Y246E/Y293E mutant lacking the PBS (**Supplementary Figure S2**), induced a decrease in haptotactic migration compared to the full length GIT1-Y246E/Y293E mutant (**Figure 7C**), suggesting that binding to paxillin is required by the activated GIT1-Y246E/Y293E protein to promote motility.

The specific effects of the GIT1-Y246E/Y293E mutant on cell motility were confirmed by a distinct assay. Wound healing was performed on COS7 cells transfected with GFP alone or in combination with GFP-tagged GIT1 constructs (**Figure 7D**). Single cell tracking showed an increase in the distance covered by the cells expressing GFP-GIT1-Y246E/Y293E compared to either GFP-GIT1 or GFP transfected cells (**Figure 7E**). Quantification confirmed the specific enhancement of the euclidean distance covered by GFP-GIT1-Y246E/Y293E-positive cells compared to cells expressing either GFP or full length GIT1 (**Figure 7F**). Altogether the results show that activation of the binding of GIT1 to paxillin correlates with positive effects on cell motility.

## Conclusions

GIT1 is able to assemble a variety of molecular complexes devoted to distinct cellular functions. Given its ability to form large molecular assemblies, the GIT1 protein must be tightly regulated. In this direction, the expression of truncated GIT1 constructs including the PBS enhances cell spreading and protrusion more evidently than full length GIT1 [10], [19], [23]. On the other hand, the findings that overexpression of specific GIT1 carboxy-terminal fragments cause large intracellular membrane-bound dysfunctional aggregates [16], [33], and that GIT1 carboxy-terminal fragments are included in huntingtin aggregates from patients [34] underscore the importance of GIT1 regulation.

Previous studies suggested that GIT1 is regulated by conformational changes implicating an intramolecular mechanism [10], [23]. According to the model, the N-terminal portion of GIT1 interacts with the C-terminal segment, keeping the molecule less accessible for binding to its partners. Here, we presented new evidence supporting this model, by identifying two tyrosine residues in the amino-terminal part of the molecule that are needed for the interaction with the carboxy-terminal part of GIT1.

Different studies point to the relevance of phosphorylation in the regulation of GIT1 function: PAK-mediated phosphorylation on serine 709 enhances GIT1 binding to paxillin and positively regulates protrusion [28], while in neurons GIT1 phosphorylation on Y392 by Src family kinases downstream of EphB receptor mediates its translocation to synapses via Grb4 [28]. Tyrosines 246 and 293 are not major sites of phosphorylation following either v-Src expression of treatment with pervanadate [35]. Still, we found here that mutation of these sites strongly reduced the levels of tyrosine phosphorylation of the GIT1-N fragment in COS7 cells overexpressing c-Src and treated with pervanadate. Hyperphosphorylation though did not appear to affect the interaction of the amino-terminal GIT1-N fragment including the two tyrosines, with the carboxy-terminal part of GIT1, nor did it increase the binding of wildtype GIT1 to paxillin, suggesting that GIT1-N tyrosine phosphorylation may not be sufficient to regulate these events. On the other hand further analysis of the stoichiometry of phosphorylation of the tyrosines 246 and 293 is required to better define the role of their post-translational modification in the activation of GIT1.

How the intramolecular interactions mediated by Y246 and Y293 can be released to activate GIT1 binding to paxillin remains an open question. One possibility is that the activation of GIT1 may rely on the binding of regulatory proteins that affect its conformation. In this direction, PAK is a suitable candidate that interacts indirectly with GIT1 via PIX [36]. PAK is required for the kinase-independent recruitment of the GIT/PIX complex via paxillin at sites of adhesion to the extracellular matrix [16], [19], [37]. A PAK mutant unable to bind PIX blocks the localization of the complex to the adhesion sites [18], while a small PAK1 PIX-binding fragment [33] is sufficient to enhance GIT1 binding to paxillin [23]. Future analysis is needed to define the mechanism by which PAK may unleash the intramolecular mechanism that keeps GIT1 inactive.

## Supporting Information

Figure S1 **Alignment of GIT1 protein sequences. The two human (NP 001078923.1, NP 054749.2), the chicken (NP 989627.1) and the mouse (NP 001004144.1) GIT1 reference sequences from NCBI databases were aligned using ClustalW.** The two tyrosines corresponding to the residues 246 and 293 of the human GIT1 protein (NP 001078923.1) are indicated.
(TIF)

Figure S2 **COS7 cells were transfected with either full length GFP-GIT1-EE or GFP-GIT1-EE-ΔPBS lacking the carboxy-terminal PBS region required for the interaction with paxillin.** Lysates were immunoprecipitated with anti-GFP. Filters with immunoprecipitates (150 μg of protein lysate, IP) and lysates (20 μg) were incubated with anti-GFP and anti-paxillin antibodies, respectively.
(TIF)
