# Supplementary figures and images for: Identification of Two Tyrosine Residues Required for the Intramolecular Mechanism Implicated in GIT1 Activation
Source: PLoS One. 2014 Apr 3;9(4):e93199. doi: 10.1371/journal.pone.0093199 (PMC3974724; doi:10.1371/journal.pone.0093199)

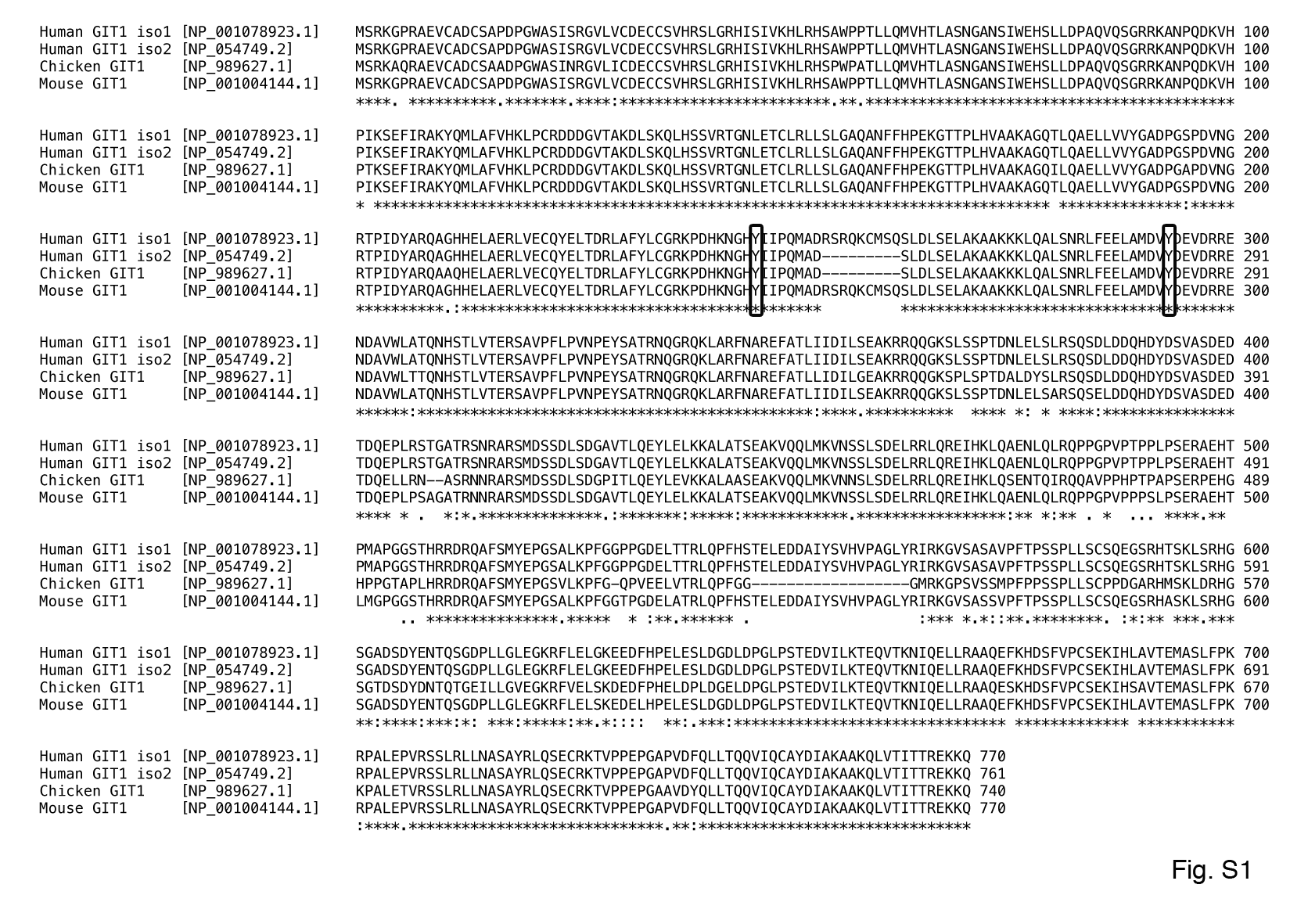

Supplement: Figure S1 — Alignment of GIT1 protein sequences. The two human (NP 001078923.1, NP 054749.2), the chicken (NP 989627.1) and the mouse (NP 001004144.1) GIT1 reference sequences from NCBI databases were aligned using ClustalW. The two tyrosines corresponding to the residues 246 and 293 of the human GIT1 protein (NP 001078923.1) are indicated. (TIF) [file pone.0093199.s001.tif]

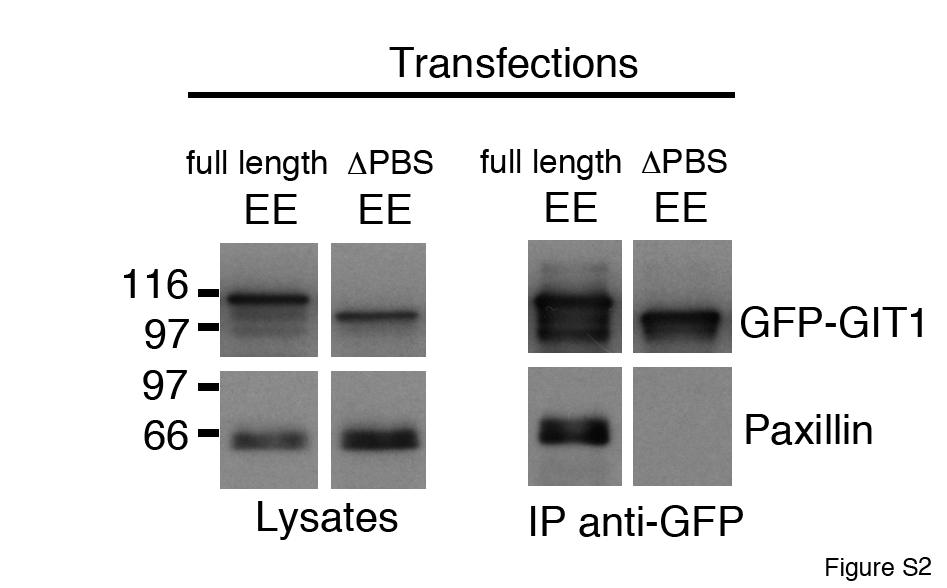

Supplement: Figure S2 — COS7 cells were transfected with either full length GFP-GIT1-EE or GFP-GIT1-EE-ΔPBS lacking the carboxy-terminal PBS region required for the interaction with paxillin. Lysates were immunoprecipitated with anti-GFP. Filters with immunoprecipitates (150 μg of protein lysate, IP) and lysates (20 μg) were incubated with anti-GFP and anti-paxillin antibodies, respectively. (TIF) [file pone.0093199.s002.tif]
